# Myeloperoxidase Negatively Regulates Neutrophil–Endothelial Cell Interactions by Impairing αMβ2 Integrin Function in Sterile Inflammation

**DOI:** 10.3389/fmed.2018.00134

**Published:** 2018-05-04

**Authors:** Alan Tseng, Kyungho Kim, Jing Li, Jaehyung Cho

**Affiliations:** ^1^Department of Pharmacology, University of Illinois at Chicago College of Medicine, Chicago, IL, United States; ^2^Korean Medicine-Application Center, Korea Institute of Oriental Medicine, Daegu, South Korea

**Keywords:** neutrophil, myeloperoxidase, αMβ2 integrin, vascular inflammation, intravital microscopy

## Abstract

Interactions of neutrophils with endothelial cells (ECs) and platelets contribute to tissue damage and vascular occlusion under sterile inflammatory conditions. However, the molecular mechanisms regulating the cell–cell interactions remain poorly understood. Previous studies suggest that reactive oxygen species, such as hydrogen peroxide (H_2_O_2_), produced from NADPH oxidase 2 play a critical role in platelet–neutrophil interactions by regulating the function of neutrophil αMβ2 integrin during sterile inflammation. In this study, we further demonstrate a crucial role for myeloperoxidase (MPO) in regulating the adhesive function of neutrophils through αMβ2 integrin. Using real-time fluorescence intravital microscopy and *in vitro* assays, we showed that loss of MPO promoted neutrophil–EC interactions and neutrophil emigration but did not affect neutrophil–platelet interactions under inflammatory conditions. Using genetic and pharmacologic approaches, we found that following agonist stimulation, MPO knockout (KO) neutrophils exhibited a significant increase in extracellular H_2_O_2_ and surface level of αMβ2 integrin and that these effects were dependent on MPO activity. Our *in vivo* studies using an ischemia/reperfusion-induced hepatic inflammation model revealed that compared to wild-type mice, neutrophils from MPO KO mice—displayed a pro-migratory phenotype while ameliorating tissue damage. These results suggest that MPO plays a negative role in the adhesive and migratory function of neutrophils by impairing αMβ2 integrin function under sterile inflammatory conditions.

## Introduction

Neutrophils are indispensable for innate immunity. However, their excessive recruitment to sites of inflammation causes severe and progressive tissue damage (1). Hence, a better understanding of the mechanisms regulating proinflammatory functions of neutrophils will help design new strategies for the treatment of inflammatory diseases. Following tissue injury, neutrophils are the first responders of host-defense against invading micropathogens. For the innate immune response, neutrophils are recruited to the site of infection. Initial neutrophil rolling over the inflamed endothelium is mediated by the interaction between selectins and their ligands (1). Subsequently, activated integrins, mainly αLβ2 and αMβ2, bind to their ligands such as intercellular adhesion molecule 1, leading to neutrophil adhesion, crawling, and transmigration. In particular, neutrophil transmigration across the endothelial cell (EC) barrier is regulated by the interactions of neutrophil integrins and other surface molecules, such as platelet endothelial cell adhesion molecule-1 (PECAM-1) and CD99, with EC junctional molecules, including junctional adhesion molecules, PECAM-1, and CD99L2 (1). The emigrated neutrophils move across the basement membrane to phagocytose microbes and release proinflammatory cytokines. Under sterile inflammatory conditions, adherent and crawling neutrophils also interact with platelets, inducing thrombus formation and contributing to microvessel occlusion (2, 3).

Among many molecules produced or released by activated neutrophils, reactive oxygen species (ROS) are crucial for the innate immune response and proinflammatory functions of neutrophils (4). Activated NADPH oxidase 2 (NOX2) is a major source for the generation of superoxide anion $\left({\text{O}}_{2}^{•-}\right)$ and is responsible for the respiratory burst in neutrophils (5, 6). Subsequently, superoxide dismutase catalyzes the dismutation of ${\text{O}}_{2}^{•-}$ to H_2_O_2_. ROS and H_2_O_2_ oxidize lipids and proteins, thereby inducing pathological signals in vascular disease (7). Myeloperoxidase (MPO), a myeloid-specific enzyme released from primary (azurophilic) granules into both the extracellular space and the phagolysosomal compartment, uses H_2_O_2_ to produce numerous oxidants including hypochlorous acid (HOCl) (8). In particular, this enzyme is abundantly expressed in neutrophils, constituting 5% of their total protein content (9). Using NOX2 knockout (KO) mice and ROS inhibitors, our lab recently demonstrated that ROS/H_2_O_2_ generated from neutrophil NOX2 play a crucial role in the heterotypic interaction between neutrophils and platelets by promoting the ligand-binding activity of αMβ2 integrin under sterile inflammatory conditions. Importantly, we found that NOX2 deletion decreases the cell–cell interactions on vessels in tumor necrosis factor-α (TNF-α)-induced vascular inflammation and attenuates tissue damage in sterile hepatic inflammation induced by ischemia/reperfusion (I/R) injury (3). These results indicate the contribution of NOX2-generated ROS to the pathogenesis of sterile inflammation. However, it remains unexplored whether MPO contributes to sterile inflammation by regulating the neutrophil-platelet heterotypic interaction.

Myeloperoxidase has been reported to play a role in the pathophysiology of sterile inflammation such as renal I/R injury (10), stroke (11), and myocardial infarction (12). Nevertheless, there are many conflicting reports showing the differential roles of MPO and MPO-generated oxidants in infectious models. For example, loss of MPO upregulates the expression of proinflammatory cytokines in neutrophils, resulting in a proinflammatory phenotype in response to zymosan, a toll-like receptor 2 agonist (13), whereas MPO KO mice challenged with lipopolysaccharide, a toll-like receptor 4 agonist, result in the opposite (14). In addition, the role of extracellular MPO has been controversial. Previous studies suggested that secreted extracellular MPO is inactive (15) and that the catalytic activity-independent function of MPO delays neutrophil apoptosis and enhances neutrophil activation (16, 17). By contrast, other studies report a function for active MPO released into the vasculature (18, 19). These results suggest that the role of extracellular MPO in neutrophil function and the pathogenesis of inflammation may depend on the disease condition.

In this study, we investigated the role of extracellular MPO in regulating neutrophil–platelet interactions in sterile inflammation. Using genetic and pharmacologic approaches, we found that MPO released from activated neutrophils does not affect the neutrophil–platelet interaction but negatively regulates αMβ2 integrin function and neutrophil–EC interactions by consuming extracellular H_2_O_2_. Our results suggest the importance of the activity-dependent function of MPO in regulating sterile. inflammation and provide evidence for the complex roles of ROS in the adhesive function of neutrophils.

## Materials and Methods

### Reagents

Human thrombin, prostaglandin E1, citrate-dextrose solution, 2% gelatin type B, 3, 3′,5,5′-tetramethylbenzidine liquid substrate, naphthol AS-D chloroacetate (esterase) kit, *N*-formyl-methionyl-leucyl-phenylalanine (fMLP), phorbol 12-myristate 13-acetate (PMA), and a protease inhibitor cocktail were purchased from Sigma (St. Louis, MO, USA). The Amplex UltraRed hydrogen peroxide and the EnzChek Gelatinase/Collagenase assay kits and Hank’s balanced salt solution (HBSS) were from Thermo Fisher Scientific (Waltham, MA, USA). Recombinant human MPO, recombinant human and mouse TNF-α, and an antibody against MPO were obtained from R&D systems (Minneapolis, MN, USA). 4-aminobenzoic acid hydrazide (4-ABAH), a specific inhibitor of MPO, was purchased from Cayman Chemical (Ann Arbor, MI, USA). Percoll was from GE Healthcare Bio-Sciences AB (Uppsala, Sweden). Nycoprep 1.077 was purchased from Axis-Shield PoC-AB (Oslo, Norway). RPMI1640 media was obtained from Mediatech (Manassas, VA, USA). d-Phe-Pro-Arg-chloromethyl ketone (PPACK) was from EMD Millipore (Billerica, MA, USA). Heparin (10,000 U/mL) was purchased from Frensinius Kabi (Lake Zurich, IL, USA). Vectashield was obtained from Vector Laboratories (Burlingame, CA, USA). PE-conjugated antibodies against mouse αM (M1/70), mouse PSGL1, total human αMβ2 (ICRF44) or activated human αMβ2 (CBRM1/5), an Alexa Fluor 647-conjugated anti-Ly-6G antibody, and PE-conjugated isotype control IgGs were purchased from BioLegend (San Diego, CA, USA). A DyLight 488-conjugated anti-mouse CD42c antibody was from Emfret Analytics (Eibelstadt, Germany). An anti-β-actin antibody was obtained from Novus Biologicals (Littleton, CO, USA). Polyclonal antibodies against αM and β2 were purchased from Santa Cruz Biotechnology (Santa Cruz, CA, USA). Human umbilical vein ECs (HUVECs) were obtained from ScienCell (Carlsbad, CA, USA).

### Mice

Wild-type (WT, C57BL/6) and B6.129 × 1-*Mpo^tm1Lus^*/J (MPO KO) mice with a C57BL/6 background were obtained from Jackson Laboratory (Bar Harbor, ME, USA). Mice were used at an age of 6–10 weeks old in this study. Age-matched mice of both genders were used in all studies. The University of Illinois Institutional Animal Care and Use Committee approved all animal care and experimental procedures.

### Isolation of Platelets and Neutrophils

Mouse platelets were prepared as previously described (20). Washed platelets were suspended in HEPES-Tyrode buffer (20 mM HEPES, pH 7.4, 136 mM NaCl, 2.7 mM KCl, 12 mM NaHCO_3_, 2 mM MgCl_2_, and 5.5 mM glucose) and adjusted to a density of 2 × 10^8^ cells/mL. Human blood and mouse bone marrow neutrophils were isolated as we described (21). The concentration of neutrophils was adjusted to 1 × 10^7^ cells/mL in HBSS buffer, unless otherwise stated. Approval to collect blood samples was obtained from the University of Illinois-Chicago review board in accordance with the Declaration of Helsinki.

### *In Vitro* Heterotypic Platelet–Neutrophil Aggregation

Platelet–neutrophil aggregation assays were performed as previously described (3). Platelets (2 × 10^7^) and neutrophils (1 × 10^6^) isolated from WT and MPO KO mice were labeled with Dylight 488-conjugated anti-CD42c and Alexa Fluor 647-conjugated anti-Ly-6G antibodies, respectively. Platelets were activated with 0.025 U/mL thrombin for 5 min at 37°C, followed by incubation with 50 µM PPACK. Neutrophils were mixed with activated platelets under a stirring condition of 1,000 rpm in an aggregometer. After a 5-min incubation, cells were fixed and analyzed by flow cytometry.

### Flow Chamber Assay

Confluent HUVEC monolayers on glass coverslips coated with 0.2% gelatin were stimulated with 20 ng/mL TNF-α for 6 h and placed into a parallel plate flow chamber (Bioptech) as we described previously (22). Mouse WT and MPO KO neutrophils (1 × 10^6^/mL) in HBSS-HEPES buffer (20 mM HBSS buffer supplemented with 10 mM HEPES pH 7.4, 2 mM CaCl_2_, 1 mM MgCl_2_, and 0.2% BSA) were perfused for 10 min over the activated HUVECs under venous shear (1 dyn/cm^2^), followed by real-time image acquisition using a Nikon microscope (ECLIPSE Ti, Melville, NY, USA) equipped with 10×/0.25 NA objective lens and recorded with a camera (CoolSNAP ES2). HBSS-HEPES buffer was then perfused for 5 min to wash out weakly bound neutrophils. The data were analyzed using NIS Elements (AR 3.2). Adherent neutrophils were monitored in a field of 0.15 mm^2^ and counted in 4–5 separate fields.

### Measurement of ROS Production

Isolated neutrophils, 1 × 10^5^/mL (for PMA) and 1 × 10^6^/mL (for fMLP) in HBSS buffer, were treated with different concentrations of an agonist, followed by measurement of extracellular H_2_O_2_ using the Amplex UltraRed reagent (ThermoFisher) according to the manufacturer’s protocol. The fluorescence signal was read in a FlexStation II benchtop fluorometer (Molecular Devices) with an excitation wavelength of 530 nm and an emission wavelength of 590 nm.

### MPO Activity Assay

Isolated neutrophils in HBSS buffer or phosphate-buffered saline, pH 7.4 (PBS) were pretreated with vehicle or the indicated concentrations of 4-ABAH, a specific inhibitor of MPO, for 30 min at 37°C. The cells were sonicated in PBS (8 × 10^6^ and 1 × 10^6^/mL for mouse and human neutrophils, respectively) containing protease inhibitor cocktail and 1 mM PMSF. The cell lysate was collected after centrifugation and sample reactions were prepared in duplicates. Each reaction contained the lysates of 4 × 10^5^ mouse neutrophils or 1 × 10^4^ human neutrophils with 0.3 mM H_2_O_2_ and 10.5% 3,3′,5,5′-Tetramethylbenzidine solution (ThermoFisher). The reaction was quenched with the addition of 0.8 N HCl and the absorbance was read on a PHERAstar plate reader (BMG Labtech) at 450 nm. In some experiments, 1.6 × 10^8^/mL mouse neutrophils were stimulated with or without 10 ng/mL PMA for 10 min at 37°C after pretreatment with vehicle or 500 µM 4-ABAH. The supernatant was collected and used in the MPO activity assay. In other experiments, recombinant human MPO (100 ng) was treated with vehicle or the indicated concentrations of 4-ABAH and used in the assay.

### Gelatinase Activity Assay

Neutrophils (2 × 10^7^/mL in RPMI1640 media) were stimulated with the indicated concentrations of fMLP or PMA for 10 min at 37°C. After centrifugation, the supernatant was used to measure gelatinase activity using the EnzChek^®^ Gelatinase/Collagenase Assay Kit (ThermoFisher). The fluorescence intensity was read on a PHERAstar plate reader with an excitation wavelength of 485 nm and emission wavelength of 520 nm for 4 h. The gelatinase activity was calculated at a fixed time point within the linear range of data points.

### Flow Cytometry

Neutrophils (2 × 10^6^/mL in RPMI1640 media) were treated with or without different concentrations of fMLP or PMA for 10 min at 37°C. In some experiments, 200 nM H_2_O_2_ was added immediately prior to agonist stimulation. In other experiments, cells were treated with vehicle or 500 µM 4-ABAH for 30 min at 37°C prior to agonist stimulation. For mouse neutrophils, cells were incubated with phycoerythin (PE)-conjugated antibodies against αMβ2, PSGL-1, or isotype control immunoglobulin (IgG). For human neutrophils, cells were labeled with PE-conjugated antibodies against total αMβ2 (ICRF44), activated αMβ2 (CBRM1/5), or isotype control IgG. The cells were then fixed with 1% paraformaldehyde, followed by flow cytometric analysis using a Cyan-ADP flow cytometer (Beckman Coulter). The mean or median fluorescence intensity value of antibodies were normalized to that of the respective unstimulated control and expressed as a fold increase.

### Immunoblotting

Mouse neutrophils were lysed at a concentration of 5 × 10^7^/mL in a lysis buffer (50 mM Tris–HCl, pH 7.4 containing 1% NP-40, 0.5% sodium deoxycholate, 0.1% SDS, 150 mM NaCl, 2 mM EDTA, protease inhibitor cocktail, and 1 mM PMSF) on ice. For the releasate immunoblot, mouse neutrophils (2 × 10^7^/mL in RPMI1640 media) were stimulated with the indicated concentrations of an agonist for 10 min at 37°C. The supernatant was collected after centrifugation and the cell pellets lysed with the lysis buffer at a volume equal to the volume of supernatant collected.

### Intravital Microscopy

Intravital microscopy was performed in a mouse model of TNF-α-induced cremaster venular inflammation as we described previously (2). WT and MPO KO male mice (6–8 weeks old) were anesthetized with i.p. injection of ketamine and xylazine, followed by exteriorization of the cremaster muscle. Neutrophils and platelets were visualized by infusion of Alexa Fluor 647-conjugated anti-Ly-6G [0.05 µg/g body weight (BW)] and DyLight 488-conjugated anti-CD42c (0.1 µg/g BW) antibodies, respectively. Images were captured in 10 different cremaster venules with a diameter of 25–40 µm in one mouse and recorded using an Olympus BX61W microscope with a 60 × 1.0 NA water immersion objective and a high-speed camera (ORCA-Flash4.0 V2, C11440, Hamamatsu). The numbers of rolling and adherent neutrophils were determined in an area of 0.02 mm^2^ over 5 min, followed by normalization to vessel length. The kinetics of platelet accumulation was monitored by the integrated median fluorescence intensity of the anti-CD42c antibody. The fluorescence signal was normalized to the number of adherent neutrophils and the vessel length and plotted as a function of time. Time “0” was set to when image capture began on each vessel. Data were analyzed using Slidebook (version 6.0, Intelligent Imaging Innovations).

### Hepatic I/R Injury

Hepatic I/R injury was induced as we previously described (3). WT and MPO KO mice were anesthetized with ketamine and xylazine and placed on a temperature-controller to maintain the body temperature at 37°C. The liver was exposed with a midline incision. A vascular clamp was placed on the portal vein and hepatic artery caudal to the porta hepatis for 30 min. The clamp was then removed, and the incised abdomen was covered with gauze soaked in sterile saline. Three hours after reperfusion, blood was drawn from the inferior vena cava, and the median and left lobes of the liver were taken out. The damage of hepatocytes was determined by measuring serum levels of aspartate aminotransferase. The median lobe was fixed in 10% formalin for the paraffin-embedded sections. The liver sections were stained with naphthol ASD-chloroacetate esterase kit (Sigma). Images were taken using a Zeiss Axioplan 2 microscope (Carl Zeiss, Oberkochen, Germany) with a 20 × 0.5 NA objective and an Axiocam MRc5 camera. The number of transmigrated neutrophils was counted outside the hepatic vessels.

### Statistics

Data were analyzed with the GraphPad Prism 7 software (v7.04). Statistical significance was assessed by Student’s *t-*test or one- or two-way analysis of variance (ANOVA) and Tukey’s test. A *P* value less than 0.05 was considered significant.

## Results

### Loss of MPO in Mice Decreases Neutrophil Adhesion to Inflamed Endothelium and Neutrophil–Platelet Interactions but Enhances Neutrophil Extravasation During Vascular Inflammation

**Figure 1:**
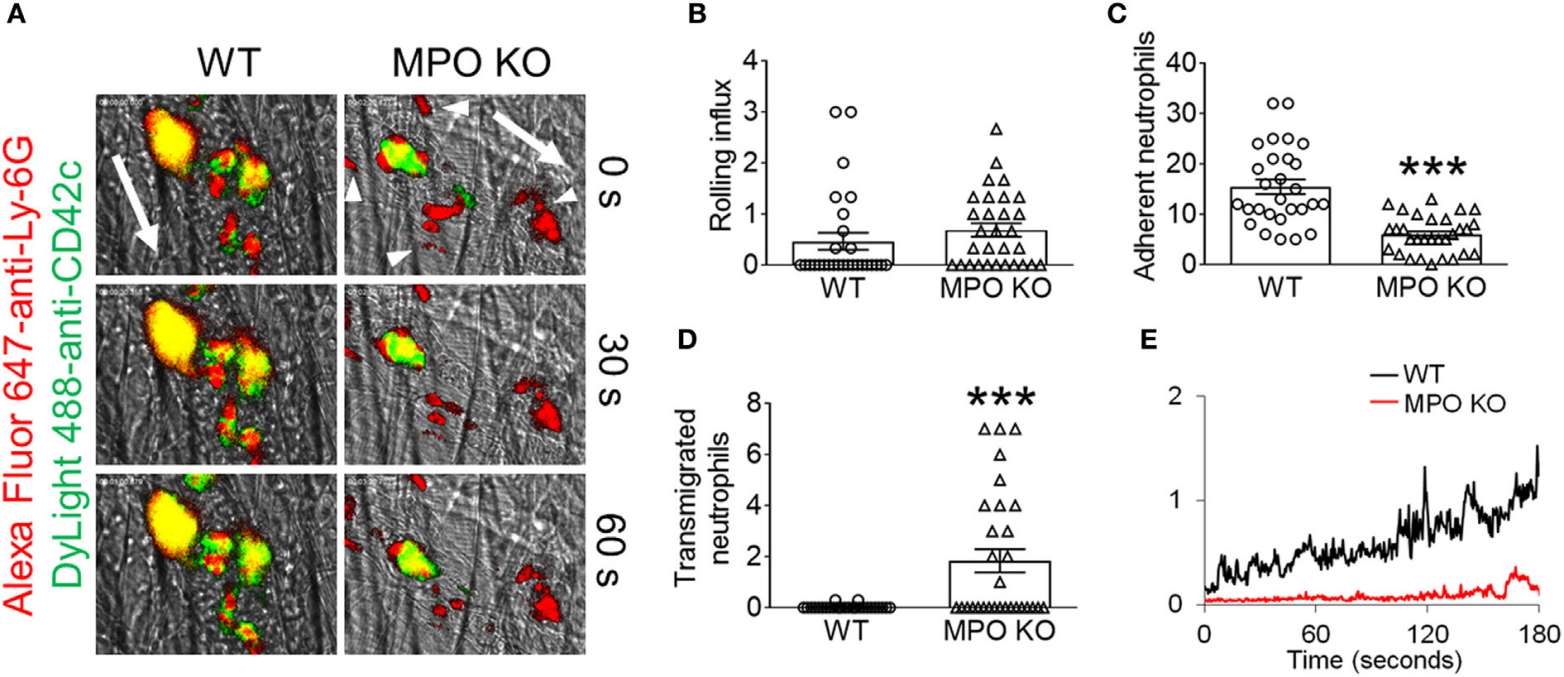
Loss of myeloperoxidase (MPO) increases neutrophil transmigration in sterile vascular inflammation *in vivo*. Intravital microscopy was performed as described in “Materials and Methods.” Intrascrotal injection of tumor necrosis factor-α induced cremaster vascular inflammation, and neutrophils and platelets were labeled by infusion of Alexa Fluor 647-conjugated anti-Ly-6G and DyLight 488-conjugated anti-CD42c antibodies, respectively. **(A)** Representative images at various time points. The direction of blood flow is indicated by the arrows. Arrowheads highlight emigrated neutrophils. **(B)** The rolling influx of neutrophils (cells per minute). **(C)** The number of adherent neutrophils (cells per 5 min). **(D)** The number of transmigrated neutrophils (cells per 5 min). **(E)** The platelet signal (integrated median fluorescence intensities of anti-CD42c antibodies) over time was quantified and normalized to the number of adherent neutrophils and the vessel length. Data are shown as the mean ± SEM (*n* = 30 venules from 3 mice per group). ***P < 0.001 versus wild-type (WT) after Student’s *t*-test.

Our previous work in NOX2 KO mice demonstrated a crucial role for NOX2-generated ROS in mediating neutrophil–platelet interactions during TNF-α-induced vascular inflammation. To examine the role of MPO in neutrophil recruitment and platelet–neutrophil interactions, we assessed MPO KO mice in the same TNF-α-induced venular inflammation model. Compared to WT mice, MPO KO mice did not exhibit any defect in neutrophil rolling over the inflamed endothelium (Figures 1A,B; Videos S1 and S2 in Supplementary Material). Deletion of MPO significantly reduced the number of neutrophils adherent to the inflamed endothelium but increased the number of transmigrated neutrophils across the endothelial barrier (Figures 1C,D). When normalized to the number of adherent neutrophils, the platelet signal was decreased in MPO KO mice as compared with WT mice (Figure 1E). In a control experiment, MPO deletion did not affect blood counts (Table 1). Since deletion of hematopoietic cell NOX2 results in a remarkable decrease in platelet–neutrophil interactions without affecting neutrophil adhesion to activated ECs (3), these results indicate that neutrophil MPO plays a distinct role in neutrophil recruitment and platelet–neutrophil interactions during vascular inflammation.

**Table 1:** The number of circulating blood cells in WT and MPO KO mice.

	WBC (10^3^/μL)	NE (10^3^/μL)	LY (10^3^/μL)	MO (10^3^/μL)	RBC (10^6^/μL)	PLT (10^3^/μL)	MPV (fL)
WT	6.5 ± 0.8	1.1 ± 0.3	5.2 ± 0.6	0.1 ± 0.1	8.1 ± 1.2	966.3 ± 215	4.4 ± 0.4
MPO KO	5.9 ± 1.5	0.9 ± 0.3	4.9 ± 1.3	0.2 ± 0.1	8.2 ± 1.1	973.8 ± 136	4.6 ± 0.4

*Blood cells from WT and MPO KO mice were counted using an automated hematology analyzer (Hemavet 950, Drew Scientific). Data represent the mean ± SD (*n* = 8 mice per group)*.

*WBC, white blood cells; NE, neutrophils; LY, lymphocytes; MO, monocytes; RBC, red blood cells; PLT, platelets; MPV, mean platelet volume; WT, wild-type; MPO, myeloperoxidase; KO, knockout*.

### Neutrophil MPO Negatively Regulates Neutrophil–EC Interactions but Does Not Affect Neutrophil–Platelet Interactions Under Inflammatory Conditions

**Figure 2:**
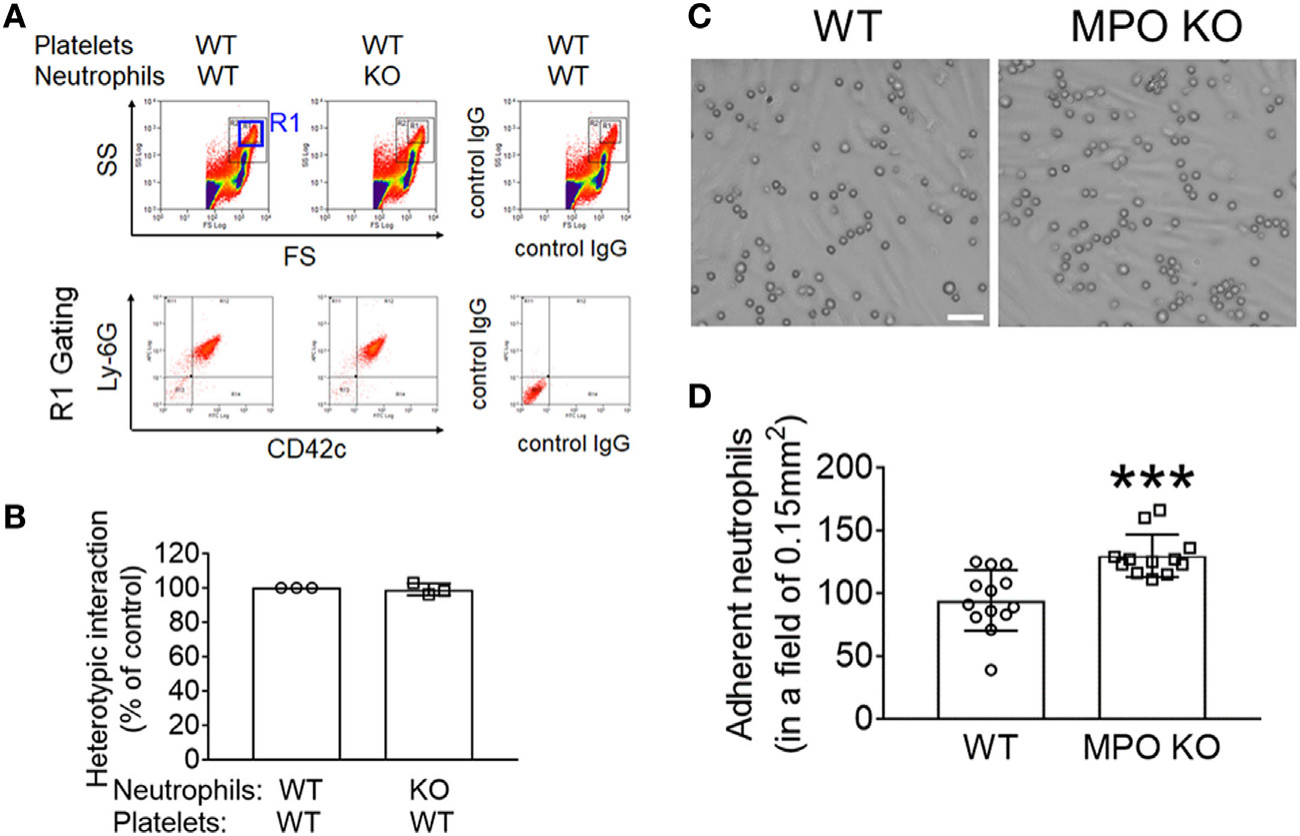
Myeloperoxidase (MPO) deficiency increases neutrophil adhesion to the inflamed endothelium without affecting platelet–neutrophil interactions *in vitro*. **(A,B)** *In vitro* neutrophil–platelet aggregation assay was performed using neutrophils and platelets isolated from wild-type (WT) and MPO knockout (KO) mice as described in Section “Materials and Methods.” **(A)** Representative histograms are shown. **(B)** Neutrophil–platelet aggregation was measured by the fluorescence intensity of anti-CD42c antibodies in the R1 gate (platelet–neutrophil aggregates) and expressed as percentage of the control. **(C,D)** Isolated WT and MPO KO neutrophils were perfused over a tumor necrosis factor-α -stimulated human umbilical vein EC monolayer under 1 dyn/cm^2^, followed by real-time microscopy. **(C)** Representative images were taken after 10 min of perfusion and 5 min of buffer only perfusion to remove weakly bound neutrophils and were cropped. Bar = 30 µm. **(D)** Quantification of **(C)**. The number of adherent neutrophils counted in a field of 0.15 mm^2^. Data are shown as the mean ± SD (*n* = 12–13 images from 3 mice per group). ***P < 0.001 versus WT after Student’s *t*-test.

To directly examine whether MPO affects the interaction between neutrophils and platelets, we performed the *in vitro* neutrophil–platelet aggregation assay under stirring conditions mimicking blood shear. We previously reported that activated platelets and neutrophils aggregate with each other under shear conditions, creating a new population in the R1 gate (21). As quantified by the fluorescence intensity of anti-CD42c antibodies in the R1 gate, deletion of neutrophil MPO did not affect neutrophil–platelet aggregation (Figures 2A,B). These results suggest that the decreased platelet–neutrophil interaction seen in intravital microscopy was likely derived from a significant decrease in the number of adherent neutrophils remaining in the vessel due to increased emigration.

We then investigated the contribution of MPO to neutrophil adhesion to activated ECs, which is the step prior to neutrophil transendothelial migration. To study this, we perfused WT and MPO KO neutrophils over a TNF-α-stimulated HUVEC monolayer under venous shear. Compared to WT neutrophils, MPO KO neutrophils exhibited an increased adhesion to the inflamed EC monolayer (Figures 2C,D). Altogether, our data show that MPO negatively regulates neutrophil–EC interactions.

### Neutrophil MPO Plays a Negative Role in the Membrane Translocation of αMβ2 Integrin Upon Cell Activation

**Figure 3:**
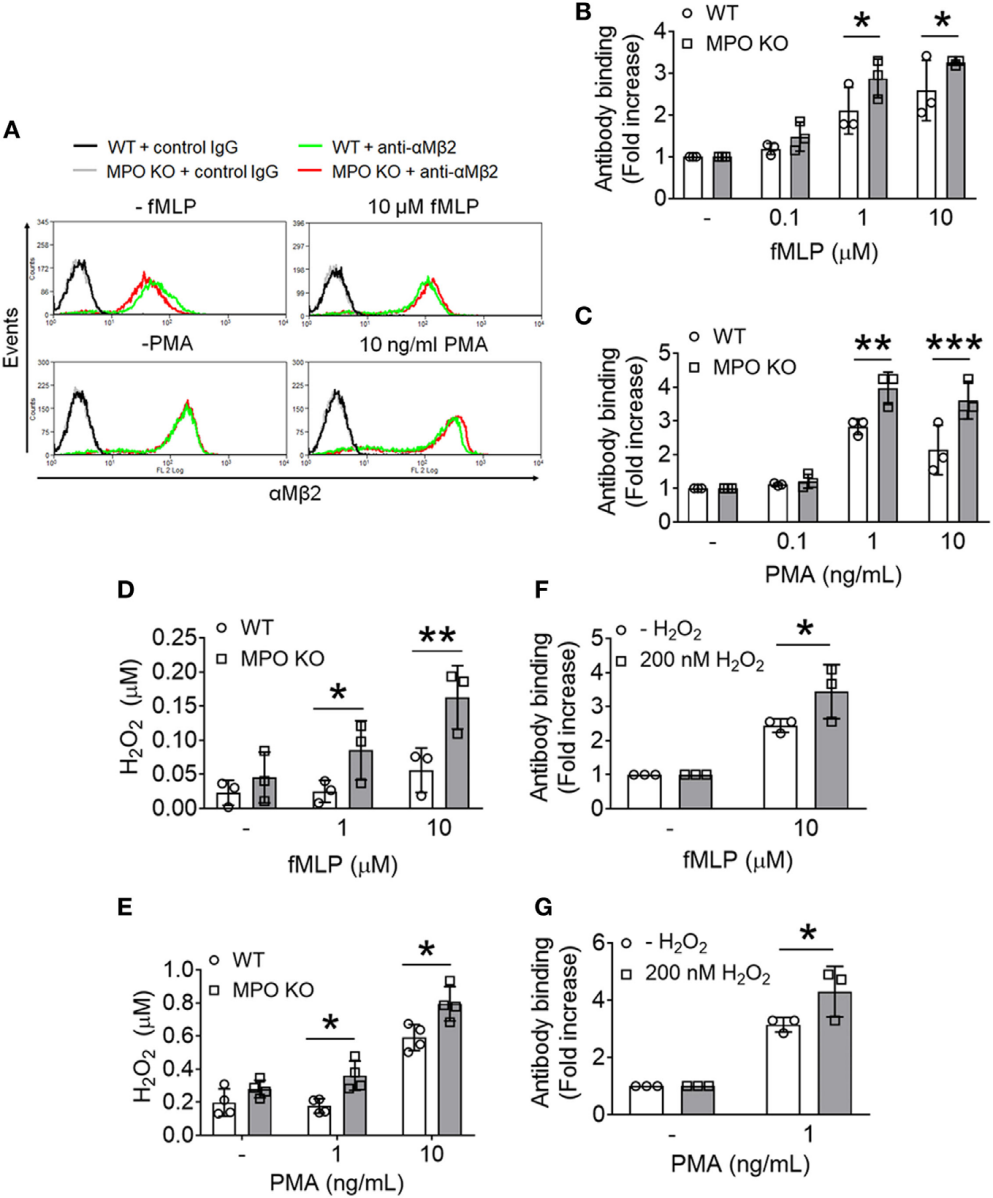
Loss of myeloperoxidase (MPO) enhances membrane translocation of αMβ2 integrin and accumulates extracellular H_2_O_2_ following agonist stimulation. Wild-type (WT) and MPO knockout (KO) neutrophils were stimulated with varying concentrations of formyl-methionyl-leucyl-phenylalanine (fMLP) or PMA. **(A–C)** Flow cytometric analysis was performed using an anti-αMβ2 antibody (M1/70). **(A)** Representative histograms. **(B,C)** Flow cytometric results are shown as a fold increase of the median fluorescence intensity relative to the respective unstimulated control. **(D,E)** Extracellular H_2_O_2_ levels were measured in fMLP- or PMA-stimulated WT and MPO KO neutrophils using the Amplex Red assay. The fluorescence signal was converted to the molar amount of H_2_O_2_ using a standard curve. **(F,G)** Exogenous H_2_O_2_ (200 nM) was added prior to stimulation of WT neutrophils with fMLP or PMA. The surface level of αMβ2 integrin was measured by flow cytometry. Data are shown as the mean ± SD (*n* = 3). *P < 0.05, **P < 0.01, or ***P < 0.001 versus WT after Student’s *t*-test.

We and others demonstrated that αMβ2 integrin is required for neutrophil–EC interactions, neutrophil transmigration, and neutrophil–platelet interactions (3, 22–24). Unlike other integrins which are mostly present on the plasma membrane, αMβ2 integrin is also stored in secondary (specific) and tertiary (gelatinase) granules and secretory vesicles (25). When neutrophils are stimulated, the integrin is translocated to the plasma membrane and activated, promoting neutrophil adhesive function. We observed that compared to WT neutrophils, MPO KO neutrophils exhibited increased surface levels of αMβ2 integrin after stimulation with different concentrations of fMLP or PMA (Figures 3A–C). There was no statistical difference of the integrin level between unstimulated WT and KO neutrophils (Figure 3A). As assessed by immunoblotting, the increased surface level of αMβ2 integrin in MPO KO neutrophils was not due to an increased expression of αMβ2 integrin protein (Figure S1A in Supplementary Material). In another control experiment, this increased surface expression is not derived from a general increased reactivity in MPO KO neutrophils since PSGL-1 shedding, another surface marker of neutrophil activation, was not increased by MPO deletion (Figure S1B in Supplementary Material). To further explore the role of MPO in degranulation, we assessed the release of gelatinase from tertiary granules. We found that treatment of neutrophils with fMLP or PMA released gelatinase (Figure S2A in Supplementary Material) and that there was no difference in the degranulation of gelatinase containing granules in WT and MPO KO neutrophils (Figures S2B,C in Supplementary Material). As a control experiment, both agonists concentration-dependently increased secretion of MPO (Figure S2D in Supplementary Material) from primary granules. These results suggest that the pro-adhesive and pro-migratory phenotype in MPO KO neutrophils may result from increased αMβ2 degranulation from granules which are different from ones containing gelatinase.

### Accumulated Extracellular H_2_O_2_ by MPO Deletion Facilitates the Membrane Translocation of αMβ2 Integrin

In the neutrophil respiratory burst, MPO generates the cytotoxic HOCl from H_2_O_2_. Recently, we demonstrated that loss of H_2_O_2_ generation in NOX2 KO neutrophils impairs the ligand-binding activity of αMβ2 integrin without affecting the membrane translocation during cell activation and that such a defect was completely rescued by the addition of 1 µM H_2_O_2_ to NOX2 KO cells (3). Since MPO deletion enhanced the surface level of αMβ2 integrin following agonist stimulation (Figures 3A–C), we hypothesized that loss of MPO accumulates H_2_O_2_ in the extracellular space. To examine this, we used the Amplex Red assay which detects extracellular H_2_O_2_ (26). Compared to WT neutrophils, MPO KO neutrophils had increased levels of extracellular H_2_O_2_ in response to fMLP or PMA (Figures 3D,E). To test whether this increase in H_2_O_2_ levels facilitates the membrane translocation of αMβ2 integrin, we added exogenous H_2_O_2_ prior to stimulation with fMLP or PMA to WT neutrophils and examined the surface levels of αMβ2 integrin. With the addition of 200 nM H_2_O_2_, WT neutrophils increased the surface level of αMβ2 integrin (Figures 3F,G). These results suggest that loss of MPO results in increased extracellular H_2_O_2_ levels upon cell activation, which facilitates the membrane translocation of αMβ2 integrin.

### The Enzyme Activity of MPO Is Responsible for the Increased Membrane Translocation and Activation of αMβ2 Integrin

Since the enzyme activity of MPO is critical for the production of HOCl from H_2_O_2_ (8), we utilized the specific MPO inhibitor, 4-ABAH (27). While 4-ABAH (1–100 µM) inhibited the activity of recombinant human MPO in a concentration-dependent manner (Figure S3A in Supplementary Material), much higher concentrations (100–500 µM) of 4-ABAH were required to inhibit MPO in mouse neutrophils (Figure S3B in Supplementary Material), which may result from poor cell permeability of the inhibitor. We observed that pretreatment with 500 µM 4-ABAH inhibited MPO activity by >80% in neutrophils. MPO KO neutrophils had no activity, demonstrating specificity of this assay in neutrophils. With pretreatment of the inhibitor, we observed >75% decrease in the activity of MPO released from PMA-stimulated neutrophils (Figure S3C in Supplementary Material), indicating that the released MPO bound to 4-ABAH did not have any activity. As a control, we confirmed that treatment with the inhibitor did not impair MPO secretion after agonist stimulation (data not shown).

**Figure 4:**
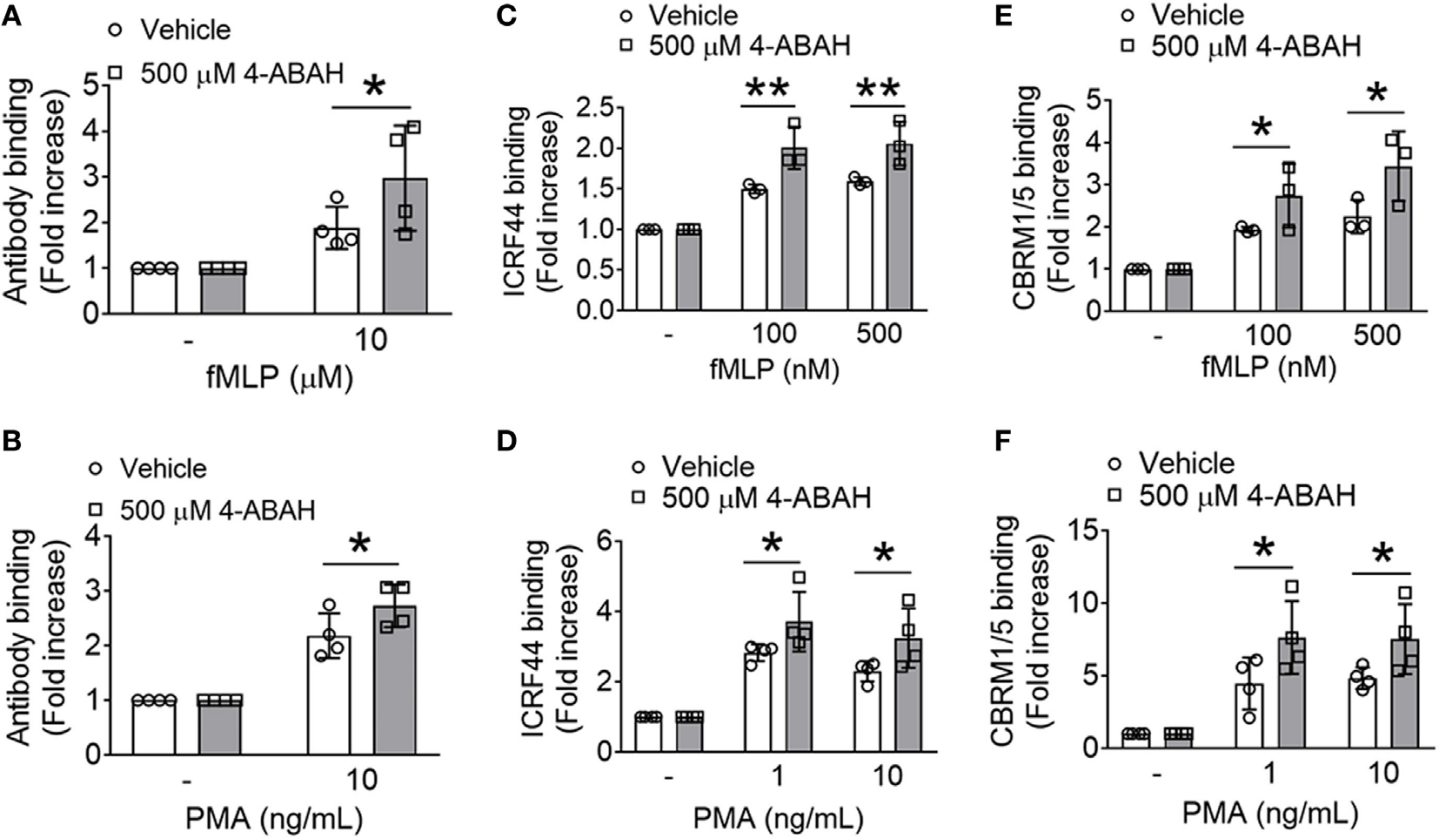
Myeloperoxidase (MPO) activity is important for the membrane translocation and activation of αMβ2 integrin. **(A,B)** Wild-type (WT) mouse neutrophils were preincubated with vehicle or 500 µM 4-ABAH, an MPO inhibitor, and stimulated with formyl-methionyl-leucyl-phenylalanine (fMLP) or PMA. The surface level of αMβ2 integrin was measured by flow cytometry. **(C–F)** Human neutrophils were preincubated with vehicle or 500 µM 4-ABAH and stimulated with fMLP or PMA, followed by flow cytometric analysis using conformation-specific antibodies against total [ICRF44 **(C,D)**] or activated human αMβ2 [CBRM1/5 **(E,F)**]. Data are shown as mean ± SD. *P < 0.05 or **P < 0.01 versus WT after Student’s *t*-test.

Pretreatment of WT neutrophils with 500 µM 4-ABAH increased the surface level of αMβ2 integrin in response to fMLP or PMA (Figures 4A,B). In a control experiment, treatment of MPO KO neutrophils with 500 µM 4-ABAH did not further increase the surface level of αMβ2 integrin following agonist stimulation (Figure S4 in Supplementary Material), suggesting that 4-ABAH is specific for MPO at the concentration used in this assay. In human neutrophils pretreated with 4-ABAH, we observed a significant increase in the surface level and activation of αMβ2 integrin following fMLP or PMA stimulation as determined by the conformation-specific antibodies against total (ICRF44) or activated αMβ2 (CBRM1/5) (Figures 4C–F). In line with our findings in MPO KO neutrophils, these results suggest that inhibition of MPO activity enhances the membrane translocation and activation of αMβ2 integrin.

### Loss of MPO in Mice Enhances Neutrophil Transmigration but Reduces I/R-Induced Hepatic Injury

**Figure 5:**
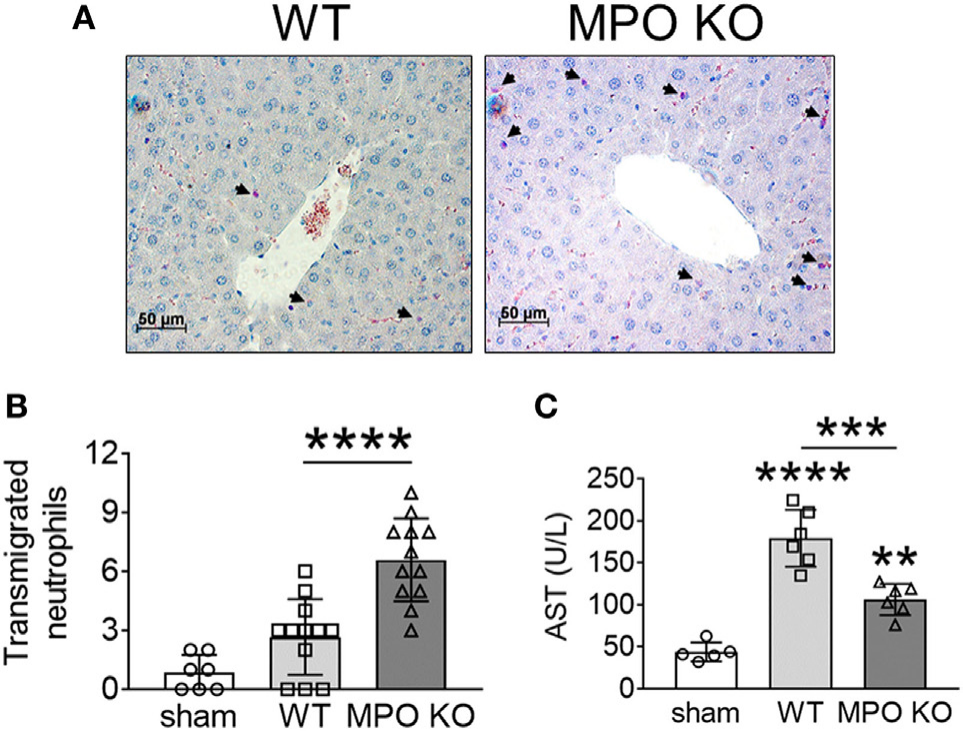
Myeloperoxidase (MPO) is important for tissue damage in I/R-induced hepatic injury. Hepatic injury was induced by I/R as described in Section “Materials and Methods.” **(A,B)** Neutrophils in the liver sections were stained with an esterase kit (pink) and counterstained with hematoxylin (purple). **(B)** The number of transmigrated neutrophils in the interstitial space. **(C)** Serum levels of aspartate aminotransferase (AST). Data are shown as the mean ± SD. **P < 0.01, ***P < 0.001, or ****P < 0.0001 after analysis of variance and Tukey’s test.

We recently reported that NOX2-generated ROS positively modulate neutrophil infiltration into damaged liver tissue, contributing to the pathogenesis of I/R-induced hepatic injury in mice (3). Thus, we sought to examine the pathophysiological role of MPO in the same disease model. We found that compared to WT mice, MPO KO mice exhibited a remarkable increase in the number of transmigrated neutrophils into the interstitial space following I/R injury (Figures 5A,B). Nevertheless, MPO deletion significantly reduced the tissue damage as assessed by serum levels of aspartate aminotransferase (Figure 5C). These results suggest that neutrophil MPO-generated oxidants, such as HOCl, are the major players in tissue damage under inflammatory conditions and support our findings that loss of MPO increases the surface level of αMβ2 integrin upon activation, which results in a pro-adhesive and pro-migratory phenotype in sterile inflammation.

## Discussion

In this study, we have found that MPO negatively regulates neutrophil adhesive function by consuming extracellular H_2_O_2_ and that loss or inhibition of MPO elevates extracellular H_2_O_2_ levels and enhances αMβ2 integrin function, facilitating neutrophil–EC interactions and subsequent neutrophil transmigration under sterile inflammatory conditions. Importantly, despite the increased transmigration of neutrophils out of the inflamed vessel, tissue damage was rather attenuated in MPO KO mice, compared to WT mice. These results indicate that MPO-produced oxidants, such as HOCl, but not H_2_O_2_, are the major player for tissue damage in sterile inflammation.

Real-time intravital microscopy has provided strong evidence that platelet–neutrophil interactions on activated ECs mediate microvascular occlusion and tissue damage under sterile inflammatory conditions (2, 3). Our recent studies using global and chimeric NOX2 KO mice revealed that hematopoietic cell NOX2 plays a critical role in neutrophil–platelet interactions but not neutrophil recruitment to the site of vascular inflammation (3). Using *in vivo* fluorescence intravital microscopy and *in vitro* assays (Figures 1 and 2), we have demonstrated that MPO deletion in mice enhances neutrophil–EC interactions and neutrophil emigration but has no significant effect on neutrophil–platelet interactions under inflammatory conditions. These results clearly show the distinct role of ROS/H_2_O_2_ versus MPO-derived oxidants in neutrophil adhesive function. A previous study using aged MPO KO mice (12–15 weeks old) suggested that MPO deletion reduces neutrophil adhesion to ECs in a manner independent of its enzymatic activity (28). However, since the authors captured only brightfield images without specific labeling of neutrophils in the microscopic study, it would be difficult to distinguish between neutrophils and other leukocytes adhered to the inflamed endothelium and to visualize transmigrated neutrophils.

The idea that MPO acts to sequester H_2_O_2_ in inflammation has been a long-standing point of contention. Early works showed increased, unchanged, or even decreased ROS production in MPO deficient neutrophils (29–31). A recent study demonstrated that loss of MPO in human neutrophils results in leakage of H_2_O_2_ into surrounding tissue with live or heat-killed *Salmonella* challenge (27). Consistently, we found that MPO deletion elevates extracellular H_2_O_2_ levels in response to fMLP or PMA (Figures 3D,E). As H_2_O_2_ is freely cell-permeable, high levels may trigger compensatory antioxidant systems, such as catalase, glutathione peroxidase, peroxiredoxins, and thioredoxins (32) and impair the activity of protein tyrosine and lipid phosphatases by oxidizing key cysteine residues (33). We previously reported that loss of ROS/H_2_O_2_ production by NOX2 deletion impairs αMβ2 integrin activation with a minimal effect on the membrane translocation and that pretreatment of NOX2 KO neutrophils with 1 µM H_2_O_2_ rescues the defect in the integrin activation (3). Our findings that either deletion or inhibition of MPO results in a significant increase in the membrane translocation of αMβ2 integrin following agonist stimulation support the importance of the enzymatic activity of extracellular MPO. Furthermore, we observed that MPO deletion accumulates 100–200 nM H_2_O_2_ after agonist treatment (Figures 3D,E) and that pretreatment of WT neutrophils with 200 nM H_2_O_2_ enhances the surface level of αMβ2 integrin (Figures 3F,G). Since loss of NOX2-generated H_2_O_2_ affects αMβ2 integrin activation but not membrane translocation, our findings suggest H_2_O_2_ in excess, due to loss of extracellular MPO, may regulate both aspects to αMβ2 integrin function (Figures 4C–F). Overall, our results suggest differential regulation of αMβ2 integrin function depending on the level of ROS generation and the cellular environment. Nevertheless, due to the numerous functions of ROS/H_2_O_2_ (7), we cannot rule out the possibility that other signaling mechanisms beyond the regulation of integrin function might be involved in the pro-migratory phenotype.

αMβ2 integrin is contained in secondary and tertiary granules as well as secretory vesicles (25). We found that there was no increase in gelatinase release in MPO KO neutrophils compared to WT neutrophils (Figures S2B,C in Supplementary Material), suggesting that the regulation of αMβ2 integrin membrane translocation could be distinct from that of tertiary granules. It has been documented that release of secretory vesicles requires less stimulus than secondary and tertiary granules (25, 34, 35). Furthermore, it has been shown that different granule types are controlled by different secretory mechanisms. For example, exocytosis of secondary and tertiary granules is regulated by VAMP-1, VAMP-2, and SNAP-23, whereas azurophilic granules by VAMP-1 and VAMP-7 (36). Rac2 was shown to be critical for primary granule release but not secondary and tertiary (37). Since our findings implicate a differential regulation of granule secretion by loss of MPO, it would be of interest to study the complex role of ROS/H_2_O_2_ and MPO-produced oxidants in granule secretion in neutrophils.

Using mouse models of TNF-α-induced vascular inflammation and I/R-induced hepatic injury, we have demonstrated that loss of MPO enhances neutrophil transmigration out of the inflamed vessels. Previously, we reported that deletion of αMβ2 integrin abrogates platelet–neutrophil interactions without impairing neutrophil adhesion to activated ECs during vascular inflammation and significantly reduces neutrophil transmigration and hepatic tissue damage induced by I/R injury (2, 3). Since our *in vitro* results show the negative effect of MPO on the adhesive function of αMβ2 integrin (Figures 3 and 4), we conclude that enhanced αMβ2 function in MPO KO neutrophils accounts for the increased neutrophil transmigration. By contrast, a study using aged MPO KO mice (12–15 weeks old) showed that MPO deletion inhibits neutrophil transmigration following I/R-induced hepatic injury (28). The discrepancy may result from the age of mice and/or a different period of ischemia and subsequent reperfusion (90 min followed by 20 h in the previous work versus 30 min followed by 3 h in our model). Although this may be another example of the complex role of MPO in different inflammatory models, our results clearly show that deletion of MPO ameliorates tissue damage in sterile inflammation. Furthermore, it has been reported that loss of MPO in sterile inflammatory models results in decreased tissue damage (10, 11, 38). This damage is likely mediated by MPO-generated oxidants such as HOCl, reactive nitrogen species such as nitrogen dioxide radical $\left({\text{NO}}_{2}^{•}\right)$, and/or tyrosyl radicals (39). It is of importance to note that unlike chronic granulomatous disease patients who are deficient in the gp91 component of the NOX2 complex and susceptible to recurrent bacterial and fungal infections (40), patients with MPO deficiency generally do not exhibit an increased frequency of infections (9). These results suggest that compared to NOX2, MPO might be an attractive therapeutic target for the treatment of numerous diseases (41, 42). Nevertheless, since MPO inhibitors are likely to increase H_2_O_2_ levels, this adverse effect should be carefully tested.

In conclusion, our studies provide mechanistic insight into how neutrophil MPO regulates the adhesive and migratory function of neutrophils and contributes to tissue damage in sterile inflammation.

## Ethics Statement

This study was carried out in accordance with the recommendations of the University of Illinois-Chicago review board. The protocol was approved by the University of Illinois-Chicago review board. All subjects gave written informed consent in accordance with the Declaration of Helsinki. This study was carried out in accordance with the recommendations of the University of Illinois Institutional Animal Care and Use Committee. The protocol was approved by the University of Illinois Institutional Animal Care and Use Committee.

## Author Contributions

AT designed and performed research, collected and analyzed data, and wrote the manuscript; KK and JL performed research and analyzed data. JC designed research, analyzed data, and wrote the manuscript.

## Conflict of Interest Statement

The authors declare that the research was conducted in the absence of any commercial or financial relationships that could be construed as a potential conflict of interest.

## Supplementary Material

Figure S1 Myeloperoxidase (MPO) deletion does not affect the expression of the integrin αM and β2 subunits and PSGL-1 shedding. **(A)** Lysates of wild type (WT) and MPO knockout (KO) neutrophils were immunoblotted. **(B)** WT and MPO KO neutrophils were treated with or without 10 µM formyl-methionyl-leucyl-phenylalanine (fMLP) or 100 ng/mL PMA. Flow cytometric analysis was performed to determine the surface level of PSGL-1. Data are shown as a fold increase of the median fluorescence intensity relative to the respective unstimulated control (mean ± SD, *n* = 4–5).

Figure S2 Degranulation of gelatinase and myeloperoxidase (MPO) in mouse neutrophils in response to formyl-methionyl-leucyl-phenylalanine (fMLP) or PMA. **(A)** Gelatinase granule release was assessed by a gelatinase activity assay in wild-type (WT) neutrophils treated with the indicated doses of fMLP or PMA. The measurement was converted to activity using a standard curve. **(B,C)** Gelatinase granule release of WT and MPO knockout (KO) neutrophils in response to fMLP or PMA treatment. **(D)** Azurophilic granule release was assessed by immunoblot of MPO in both releasate and cell pellets collected from fMLP- or PMA-treated WT neutrophils. Data are shown as the mean ± SD (*n* = 3). **P* < 0.05 or ****P* < 0.001 after analysis of variance and Tukey’s test **(A)** or Student’s *t*-test **(B,C)**.

Figure S3 The inhibitory effect of 4-ABAH on myeloperoxidase (MPO) activity. **(A)** Recombinant human MPO (rhMPO, 100 ng) was treated with vehicle or the indicated concentrations of 4-ABAH and the MPO activity was measured by the reaction with 3,3′,5,5′-Tetramethylbenzidine. **(B)** Wild-type (WT) and MPO knockout (KO) mouse neutrophils were pretreated with vehicle (0.1% DMSO) or the indicated concentrations of 4-ABAH and washed out. The neutrophils were then sonicated and MPO activity measured. **(C)** WT neutrophils were preincubated with vehicle or 500 µM 4-ABAH and treated with 10 ng/mL PMA. The supernatant was collected and used directly for the MPO activity assay. Buffer: PBS without rhMPO, cell lysate, or supernatant. Data are shown as the mean ± SD (*n* = 3–5). ****P* < 0.001 or *****P* < 0.0001 after analysis of variance and Tukey’s test.

Figure S4 The specific effect of 4-ABAH on myeloperoxidase (MPO). Wild-type (WT) and MPO knockout (KO) neutrophils were pretreated with vehicle (0.1% DMSO) or 500 µM 4-ABAH and stimulated with formyl-methionyl-leucyl-phenylalanine. The surface level of αMβ2 integrin was measured by flow cytometry using an anti-αMβ2 antibody (M1/70). Data are shown as the mean ± SD (*n* = 3). **P* < 0.05, ***P* < 0.01, or ****P* < 0.001 after two-way analysis of variance and Tukey’s test.

Videos S1–S2 Platelet–neutrophil interactions in WT and MPO KO mice during vascular inflammation. Platelets and neutrophils were visualized by infusion of DyLight 488-conjugated anti-CD42c and Alexa Fluor 647-conjugated anti-Ly-6G antibodies into WT (Video 1) and MPOKO (Video 2) mice.
