# Supplementary figures and images for: Myeloperoxidase Negatively Regulates Neutrophil–Endothelial Cell Interactions by Impairing αMβ2 Integrin Function in Sterile Inflammation
Source: Front Med (Lausanne). 2018 May 4;5:134. doi: 10.3389/fmed.2018.00134 (PMC5946029; doi:10.3389/fmed.2018.00134)

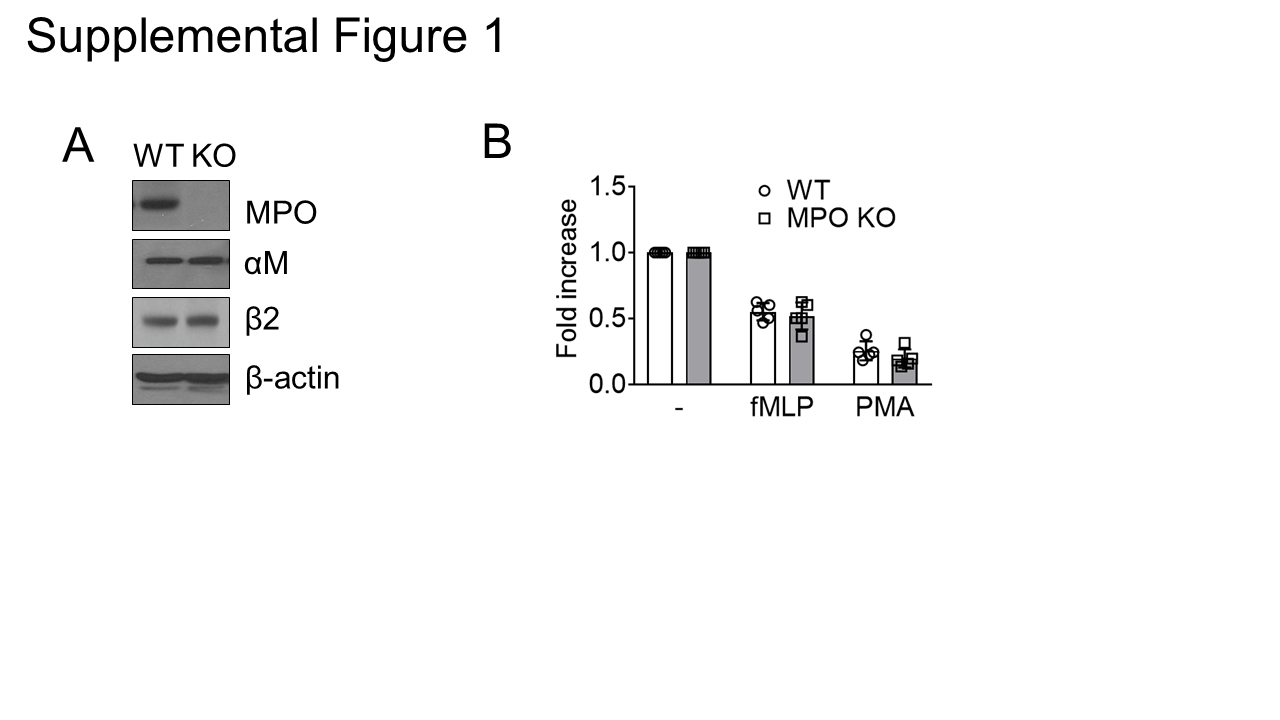

Supplement: Figure S1 — Myeloperoxidase (MPO) deletion does not affect the expression of the integrin αM and β2 subunits and PSGL-1 shedding. (A) Lysates of wild type (WT) and MPO knockout (KO) neutrophils were immunoblotted. (B) WT and MPO KO neutrophils were treated with or without 10 µM formyl-methionyl-leucyl-phenylalanine (fMLP) or 100 ng/mL PMA. Flow cytometric analysis was performed to determine the surface level of PSGL-1. Data are shown as a fold increase of the median fluorescence intensity relative to the respective unstimulated control (mean ± SD, n = 4–5). [file image_1.tif]

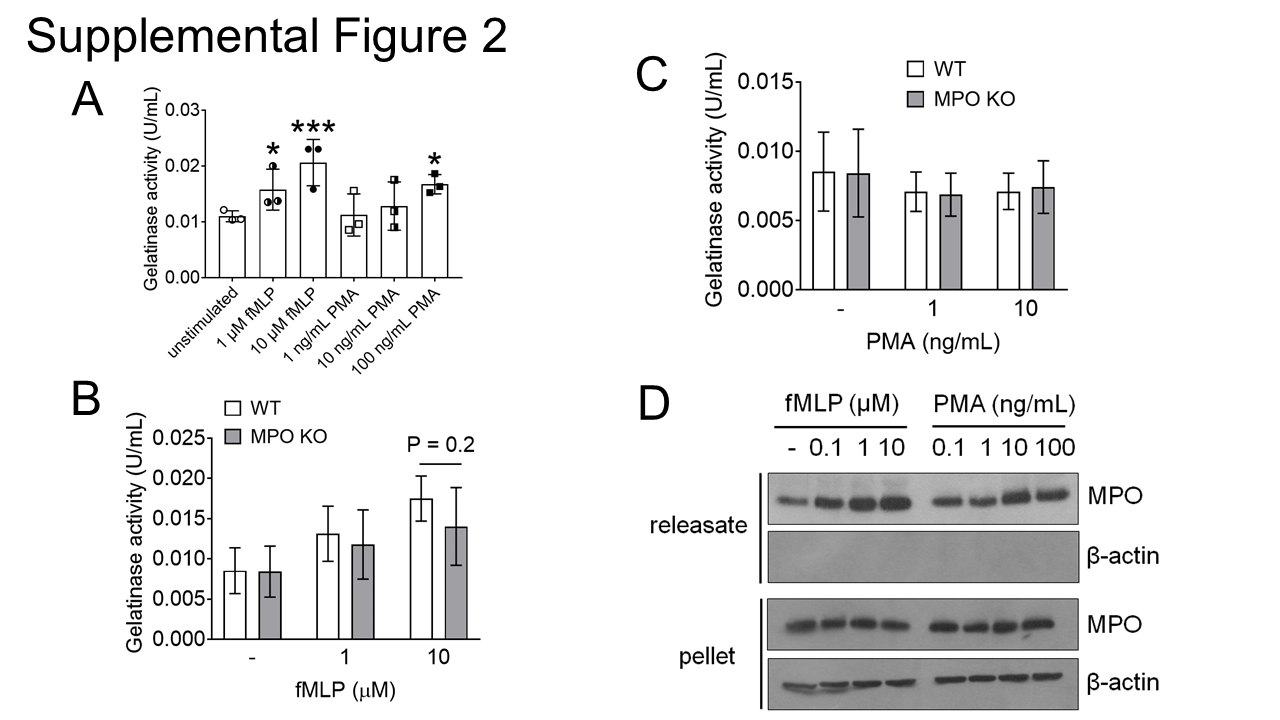

Supplement: Figure S2 — Degranulation of gelatinase and myeloperoxidase (MPO) in mouse neutrophils in response to formyl-methionyl-leucyl-phenylalanine (fMLP) or PMA. (A) Gelatinase granule release was assessed by a gelatinase activity assay in wild-type (WT) neutrophils treated with the indicated doses of fMLP or PMA. The measurement was converted to activity using a standard curve. (B,C) Gelatinase granule release of WT and MPO knockout (KO) neutrophils in response to fMLP or PMA treatment. (D) Azurophilic granule release was assessed by immunoblot of MPO in both releasate and cell pellets collected from fMLP- or PMA-treated WT neutrophils. Data are shown as the mean ± SD (n = 3). *P < 0.05 or ***P < 0.001 after analysis of variance and Tukey’s test (A) or Student’s t-test (B,C). [file image_2.tif]

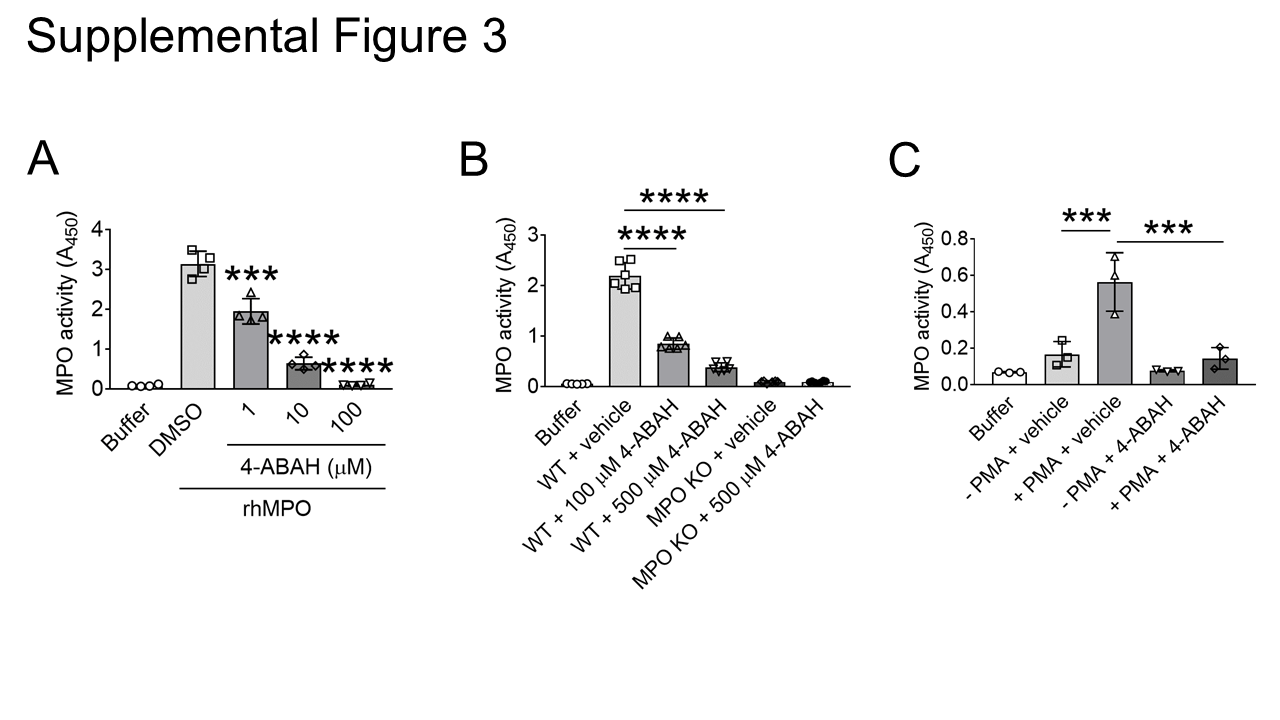

Supplement: Figure S3 — The inhibitory effect of 4-ABAH on myeloperoxidase (MPO) activity. (A) Recombinant human MPO (rhMPO, 100 ng) was treated with vehicle or the indicated concentrations of 4-ABAH and the MPO activity was measured by the reaction with 3,3′,5,5′-Tetramethylbenzidine. (B) Wild-type (WT) and MPO knockout (KO) mouse neutrophils were pretreated with vehicle (0.1% DMSO) or the indicated concentrations of 4-ABAH and washed out. The neutrophils were then sonicated and MPO activity measured. (C) WT neutrophils were preincubated with vehicle or 500 µM 4-ABAH and treated with 10 ng/mL PMA. The supernatant was collected and used directly for the MPO activity assay. Buffer: PBS without rhMPO, cell lysate, or supernatant. Data are shown as the mean ± SD (n = 3–5). ***P < 0.001 or ****P < 0.0001 after analysis of variance and Tukey’s test. [file image_3.tif]

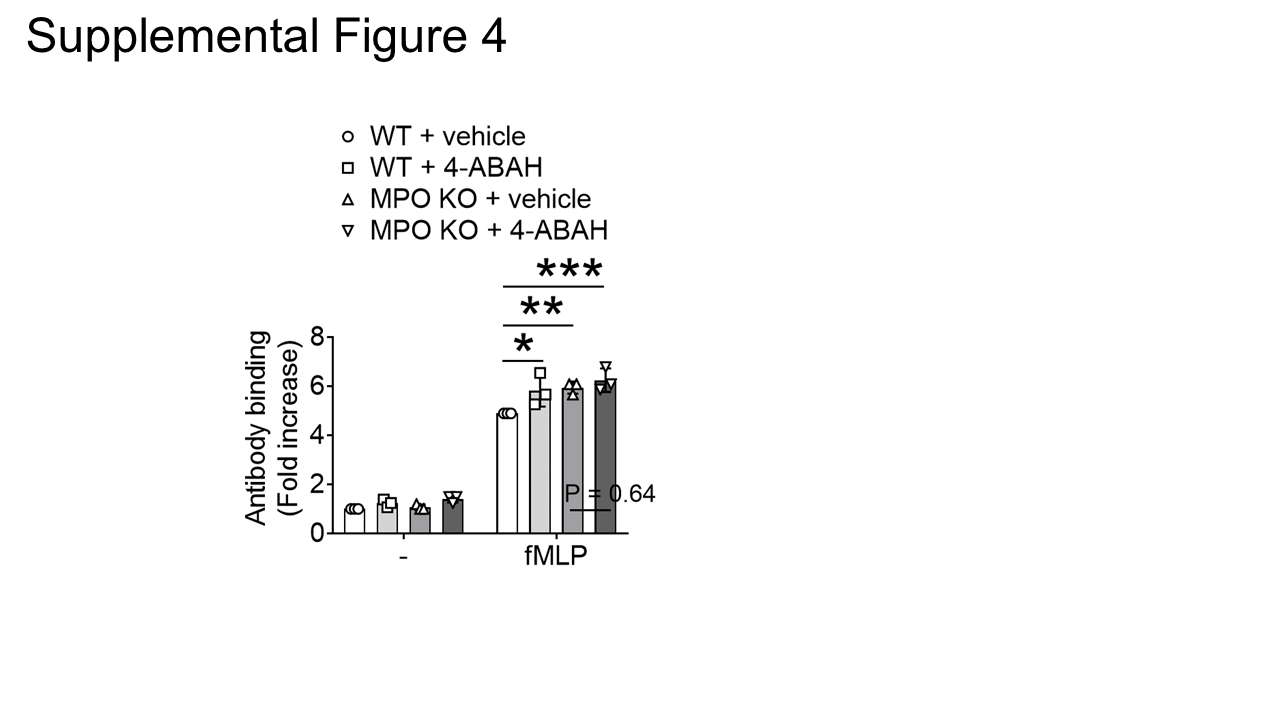

Supplement: Figure S4 — The specific effect of 4-ABAH on myeloperoxidase (MPO). Wild-type (WT) and MPO knockout (KO) neutrophils were pretreated with vehicle (0.1% DMSO) or 500 µM 4-ABAH and stimulated with formyl-methionyl-leucyl-phenylalanine. The surface level of αMβ2 integrin was measured by flow cytometry using an anti-αMβ2 antibody (M1/70). Data are shown as the mean ± SD (n = 3). *P < 0.05, **P < 0.01, or ***P < 0.001 after two-way analysis of variance and Tukey’s test. [file image_4.tif]
